# Integrating rare genetic variants into pharmacogenetic drug response predictions

**DOI:** 10.1186/s40246-018-0157-3

**Published:** 2018-05-25

**Authors:** Magnus Ingelman-Sundberg, Souren Mkrtchian, Yitian Zhou, Volker M. Lauschke

**Affiliations:** Department of Physiology and Pharmacology, Section of Pharmacogenetics, Karolinska Institutet, SE-171 77 Stockholm, Sweden

**Keywords:** Pharmacogenetics, Personalized medicine, ADME genes, Genetic variability, Drug response

## Abstract

**Background:**

Variability in genes implicated in drug pharmacokinetics or drug response can modulate treatment efficacy or predispose to adverse drug reactions. Besides common genetic polymorphisms, recent sequencing projects revealed a plethora of rare genetic variants in genes encoding proteins involved in drug metabolism, transport, and response.

**Results:**

To understand the global importance of rare pharmacogenetic gene variants, we mapped the variability in 208 pharmacogenes by analyzing exome sequencing data from 60,706 unrelated individuals and estimated the importance of rare and common genetic variants using a computational prediction framework optimized for pharmacogenetic assessments. Our analyses reveal that rare pharmacogenetic variants were strongly enriched in mutations predicted to cause functional alterations. For more than half of the pharmacogenes, rare variants account for the entire genetic variability. Each individual harbored on average a total of 40.6 putatively functional variants, rare variants accounting for 10.8% of these. Overall, the contribution of rare variants was found to be highly gene- and drug-specific. Using warfarin, simvastatin, voriconazole, olanzapine, and irinotecan as examples, we conclude that rare genetic variants likely account for a substantial part of the unexplained inter-individual differences in drug metabolism phenotypes.

**Conclusions:**

Combined, our data reveal high gene and drug specificity in the contributions of rare variants. We provide a proof-of-concept on how this information can be utilized to pinpoint genes for which sequencing-based genotyping can add important information to predict drug response, which provides useful information for the design of clinical trials in drug development and the personalization of pharmacological treatment.

**Electronic supplementary material:**

The online version of this article (10.1186/s40246-018-0157-3) contains supplementary material, which is available to authorized users.

## Background

The response of patients to medical treatment is influenced by a variety of physiological, pathological, environmental, and genetic factors [1]. These inter-individual differences can lower treatment efficacy or manifest in adverse drug reactions (ADRs), which are estimated to cause around 6.5% of all hospital admissions [2]. Overall, the genetic makeup of a patient accounts for 20–30% of the inter-individual variability in drug response [3], but for certain clinically important drugs, such as metoprolol and torsemide, twin studies suggested genetic contributions to the variability in their pharmacokinetics of up to 90% [4].

Genetic variability in phase I and phase II enzymes, transporters, cytochrome reductases, and nuclear receptors, hereafter jointly termed pharmacogenes, can modulate drug absorption, distribution, metabolism, and excretion (ADME), thereby shaping human drug response and the risk of ADRs. Prominent examples include associations of common *TPMT* variants with hematological toxicity of 6-mercaptopurines, ultrarapid metabolism of CYP2D6 with codeine toxicity, and effects of specific *CYP2C19* polymorphisms on the response to clopidogrel or proton pump inhibitors [5]. Yet, a substantial fraction of the heritable variability in drug response cannot be explained by these common variants, suggesting that other genetic factors are important contributors. In recent years, increasing capacities and decreasing costs of next-generation sequencing (NGS) platforms have facilitated large-scale studies of genetic variation and NGS assays are becoming increasingly implemented in clinical diagnostics [6]. Importantly, NGS-based analyses revealed that over 90% of the overall genetic variability in pharmacogenes is allotted to rare genetic variants, but the impact of rare genetic variability on drug pharmacokinetics has not been systematically evaluated (Additional file 1: Table S1) [7–10].

We therefore analyzed the distribution of rare and common gene variants in the 208 clinically most relevant pharmacogenes of 60,706 unrelated individuals and leveraged these genetic variability profiles to predict the relevance of rare SNVs for the pharmacokinetics or ADR risks for several clinically important drugs of diverse therapeutic areas. Based on these analyses, we conclude that the contribution of rare genetic variants is gene- and drug-specific and can account for a substantial part of the unexplained genetic inter-individual variability in drug response. Furthermore, we highlight genes for which comprehensive NGS-based genotyping instead of candidate SNP interrogations can reveal important additional information to personalize pharmacological treatment strategies. The presented data incentivizes the consideration of rare pharmacogenetic variants for the guidance of personalized drug therapy and holds important implications for the design of clinical trials.

## Methods

### Data sources

Human sequencing data from 60,706 unrelated individuals was obtained from the Exome Aggregation Consortium (ExAC) database [11], a platform that provides summary frequency information of exonic genetic variants from 17 large-scale sequencing projects. Notably, consistency of the individual data sets is assured by reprocessing of all raw data through the same bioinformatic pipelines. We complemented the obtained variants with six non-exonic variants from the 1000 Genomes Project [12] that define CYP1A2*1C (rs2069514), CYP1A2*1F (rs762551), CYP2C19*17 (rs12248560), CYP3A4*22 (rs35599367), CYP3A5*3 (rs776746), and UGT1A1*28 (rs8175347). Variants with less than 10,000 called high-quality alleles were not considered. Novel variants were defined relative to dbSNP release 135.

### Definitions

Loss-of-function (LoF) intolerance scores were provided by Lek et al. based on the expectation-maximization algorithm [11]. In brief, low scores (< 0.1) indicate that the number of protein-truncating variants is similar to what is expected by chance, whereas high scores (> 0.9) indicate much fewer of such variants are observed than would be expected, suggesting haploinsufficiency. Aggregated functional variant frequency is defined as the sum of MAFs of all variants predicted to be deleterious. Variants with MAF ≤ 0.01 were considered as rare, and variants with MAF > 0.01 were considered as common.

### Variant analyses and computational functionality predictions

Computational algorithms mostly use evolutionary conservation as a metric to predict whether a given variant likely has functional effects. Importantly, we previously evaluated 18 current functionality prediction methods and found that their predictive performance was low for poorly conserved genes, such as cytochrome P450s [13].

Here, we therefore used a functionality prediction method that we previously developed [13]. In brief, using high-quality experimental data for 123 pharmacogenetic alleles, Zhou et al. tailored the parameterization of 18 different algorithms specifically for ADME genes and integrated the results of multiple prediction methods into an ADME-optimized prediction framework [13]. Finally, the model’s performance was validated in an independent validation cohort of additional 121 experimentally characterized variants. Overall, the model achieved 92% sensitivity and 95% specificity for loss-of-function and functionally neutral variants, respectively, thereby substantially outperforming previous computational tools on pharmacogenomics data sets.

## Results

### Analysis of the genetic landscape in 208 human pharmacogenes

**Fig. 1:**
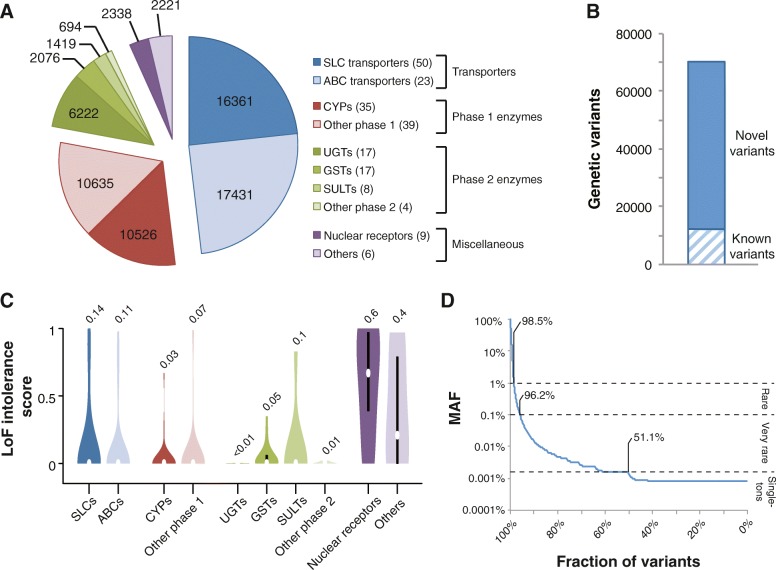
The landscape of pharmacogenomic variability. **a** Pie chart showing the distribution of the identified 69,923 variants across transporters (blue), phase 1 (red) and phase 2 (green) enzymes, and other pharmacogenes (purple). **b** 57,723 (83%) of the identified 69,923 pharmacogenetic variants were novel as compared to dbSNP release 135. **c** Violin plots showing the evolutionary constraint on loss-of-function (LoF) alleles. High scores indicate significantly less LoF variants than expected by chance. Details regarding the statistical framework are given in Lek et al. [11]. Violin plots were generated using BoxPlotR [50]. **d** Of the identified variants, 98.5 and 96.2% were rare (MAF < 1%) or very rare (MAF < 0.1%), respectively, and 51.1% of all variants were only found in a single individual

We analyzed the genetic variability in 208 genes with importance for drug ADME using exome sequencing data from 60,706 unrelated individuals. In total, we identified 69,923 variants distributed across transporter genes (33,792 variants in 73 genes), genes encoding phase 1 (21,161 variants in 71 genes) and phase 2 enzymes (10,411 variants in 46 genes), nuclear receptors (2338 variants in 9 genes), and other pharmacogenes with miscellaneous functions (2221 variants in 6 genes; Fig. 1a). Notably, 57,773 (83%) of these 69,923 variants we identified were novel as compared to dbSNP release 135 (Fig. 1b).

Evolutionary constraints in transporters as well as in phase 1 and phase 2 drug-metabolizing genes were low as judged by the large numbers of loss-of-function variants identified in these genes (LoF intolerance score = 0.08 ± 0.02 SEM; see the “Methods” section for details). In contrast, nuclear receptors and other selected genes with importance for drug response were highly LoF-intolerant (LoF intolerance score = 0.53 ± 0.11 SEM), comparable to values observed across haploinsufficient genes (LoF intolerance score > 0.5; Fig. 1c) [11]. Importantly, the vast majority of variants were rare (98.5%; MAF < 1%) or very rare (96.2%; MAF < 0.1%) and more than half (51.1%) of all variants were only detected in a single individual, highlighting the genetic diversity in human pharmacogenes (Fig. 1d).

### Rare variants contribute substantially to functional variability

**Fig. 2:**
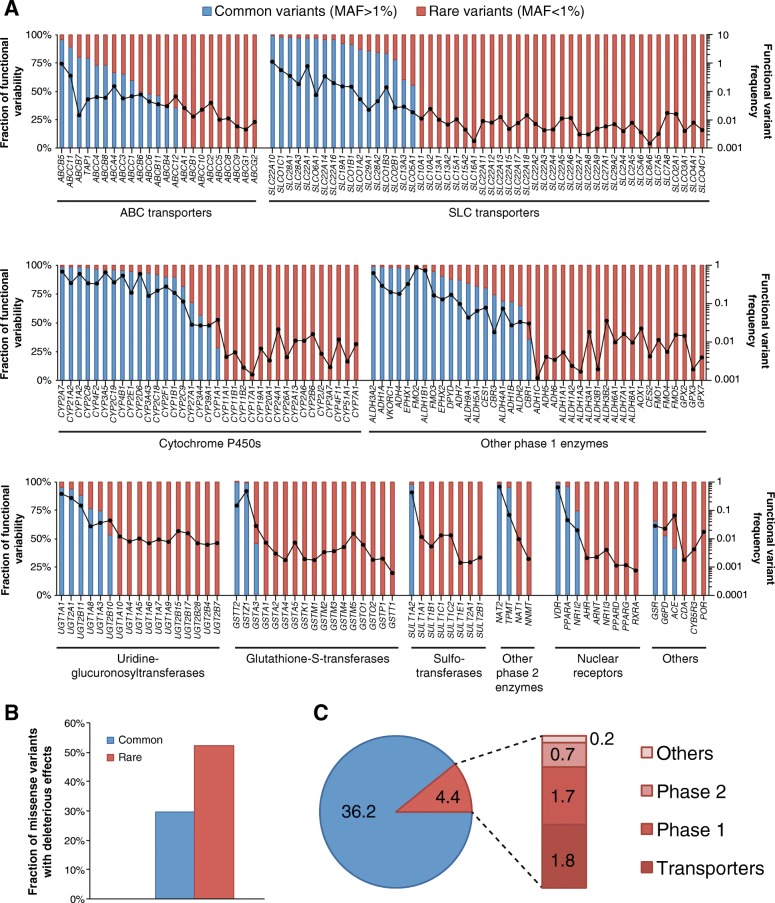
Rare genetic variants contribute substantially to pharmacogenomic variability. **a** The frequency of putatively functional variants is plotted in log scale and indicated as dots connected by the black line for each of the 208 pharmacogenes analyzed (right *y*-axis). The fraction of this functional variability that is allotted to common (blue) or rare (red) variants is indicated on the left *y*-axis. Importantly, overall genetic variability as well as the fraction of functional variation that is allotted to rare variants differs considerably between genes. **b** Rare genetic polymorphisms in pharmacogenes are enriched in variants predicted to cause functional alterations. **c** Across the 208 ADME genes analyzed, each individual was found to harbor on average 4.4 rare functional variants (frameshift, splice, start-lost, stop-gain, and putatively functional missense variants). Of these, 1.8, 1.7, 0.7, and 0.2 are allotted to transporters, phase 1 and phase 2 enzymes, and other pharmacogenes, respectively

To evaluate the functional importance of rare pharmacogenetic variants, we computed functionality assessments of each SNV using a computational assessment model specifically optimized for the assessment of pharmacogenes (see the “Methods” section for details). We then aggregated frequencies of frameshift, splice, start-lost, stop-gain, and putatively deleterious missense variants and found that the pattern and distribution of genetic variability differed substantially across the 208 pharmacogenes analyzed. Genetic variability with functional impact was governed by few high-frequency variants for some genes, including *ABCB5*, *SLC22A10*, *CYP1A2*, *CYP2C8*, or *GSTT2* (Fig. 2a). In contrast, the functionality of the majority of pharmacogenes, including *ABCB1*, *SLC10A1*, and *CYP3A7*, is dominated by rare genetic variants. The frequency of genetic variants predicted to affect the functionality of the gene product differed more than 1000-fold between genes. The most highly variable genes were *SLC22A10* (aggregated functional variant frequency 1.08), *ABCB5* (0.91), and *FMO2* (0.86), whereas the lowest numbers of functional variants were observed for *GSTT1* (0.0006), *RXRA* (0.0007), *PPARD* (0.0011), and *CYP17A1* (0.0014; Fig. 2a).

Notably, rare pharmacogenetic variants were strongly enriched in mutations predicted to cause functional alterations (Fig. 2b), consistent with previous reports [12, 14, 15]. In the 208 pharmacogenes combined, each individual harbored on average a total of 40.6 putatively functional variants (Fig. 2c). Rare variants accounted for 4.4 (10.8%) of these functional variants, of which 1.8, 1.7, 0.7, and 0.2 were allotted to transporters, phase 1, phase 2, and other pharmacogenes, respectively (Fig. 2c).

### Prediction of the importance of rare genetic variants for drug response

Given the significant contribution of rare genetic variants to the functional variability in pharmacogenes, we considered it of importance to include rare variants into predictions of drug response. Using the genetic information as template, we analyzed the contribution of rare genetic variants for drug pharmacokinetics and/or drug response, focusing on five drugs with well-characterized pharmacology and substantial unexplained inter-individual variability. Specifically, we evaluated the relevance of rare SNVs for the anticoagulant warfarin, the HMG-CoA reductase inhibitor simvastatin, the antifungal voriconazole, the antipsychotic olanzapine, and the antineoplastic agent irinotecan (Additional file 2: Table S2). We first estimated the relative importance of different genetic factors for drug metabolism phenotypes of the specific drugs based on extensive literature analysis. Subsequently in a second step, we integrated these evaluations with our genetic variability data to derive assessments of the impact of rare genetic variants on the pharmacokinetics of or response to the given drug.

#### Warfarin response

Warfarin is a racemic mixture of the R- and S-stereoisomers of which the S-form is at least five times more potent. Warfarin response is influenced by common genetic polymorphisms in *CYP2C9*, *CYP4F2*, and *VKORC1*, which jointly explain up to 45% of warfarin dose requirements [16]. Yet also other genes, such as *CYP3A4*, *CYP1A2*, *EPHX1*, and *ABCB1*, have been implicated in warfarin pharmacokinetics [17–19]. However, despite this extensive knowledge of warfarin transport and metabolism, around 40% of the variability in warfarin dose requirements remains unexplained by common genetic variants and other patient-specific factors [20].

**Fig. 3:**
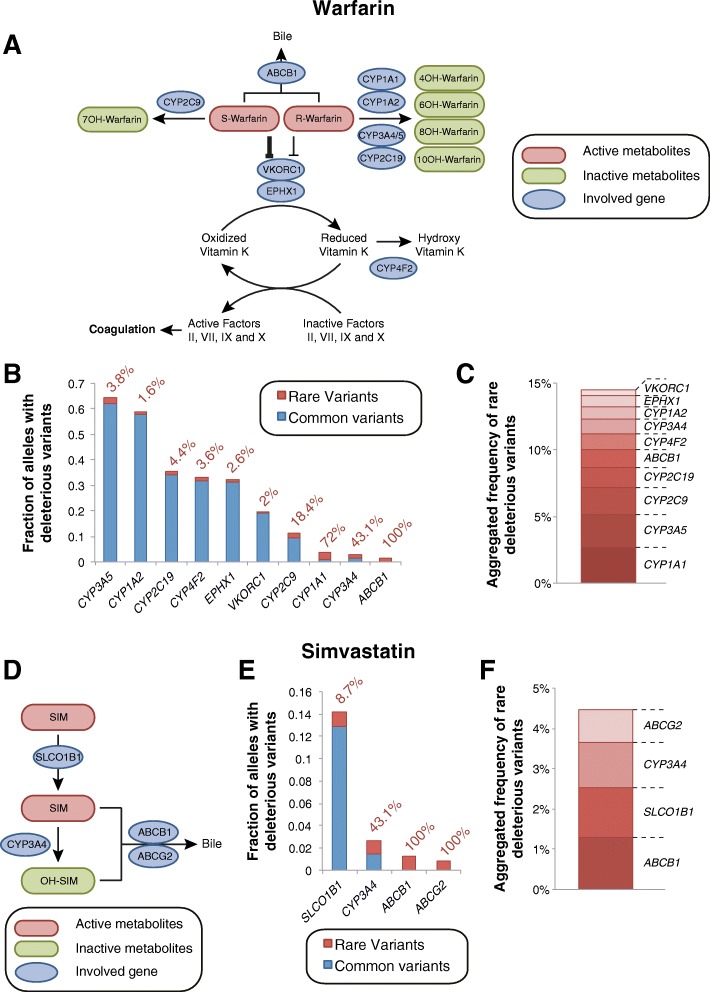
The relevance of rare genetic variants for warfarin response and simvastatin-related myotoxicity. **a** Scheme depicting the metabolism and therapeutic action of warfarin. The less potent R-enantiomer of warfarin is metabolized by CYP1A1, CYP1A2, CYP3A, and CYP2C19, whereas the more potent S-enantiomer is inactivated by CYP2C9. Warfarin inhibits the VKOR complex, which reduces vitamin K, an essential factor for the formation of functional coagulation factors. See www.pharmgkb.org/pathway/PA145011113 and www.pharmgkb.org/pathway/PA145011114 for further information. **b** Overview of the aggregated frequencies of common (MAF ≥ 1%, blue) and rare deleterious genetic variants (MAF < 1%, red) in genes involved in warfarin pharmacokinetics or pharmacodynamics. Values next to the columns indicate the relative contribution of rare genetic variants. **c** Stacked column plot showing the aggregated frequency of deleterious rare variants of potential relevance for warfarin action. **d** Scheme depicting metabolites and genetic factors involved in the hepatic uptake, metabolism, and excretion of simvastatin. See www.pharmgkb.org/pathway/PA145011109 for further information. **e** Overview of the aggregated frequencies of common (MAF ≥ 1%, blue) and rare deleterious genetic variants (MAF < 1%, red) in genes implicated in simvastatin ADME. Values next to the columns indicate the relative contribution of rare genetic variants. **f** Stacked column plot showing the aggregated frequency of deleterious rare variants of potential relevance for simvastatin pharmacokinetics

Our analyses predict that rare genetic variants contribute only minorly to the metabolism of the pharmacologically less potent R-enantiomer of warfarin (Fig. 3a–c). Similarly, their contribution to warfarin pharmacodynamics by alterations in CYP4F2 (3.6%) and VKORC1 (2%) function is expected to be relatively low. Importantly however, rare SNVs have a major impact on hepatic S-warfarin metabolism. Overall, 2.1% of *CYP2C9* alleles are predicted to harbor rare variants with deleterious effects, accounting for 18.4% of the genetically encoded functional differences in CYP2C9 activity (Fig. 3b). Moreover, our analyses predict rare variants with functional consequences in 1.3% of *ABCB1* alleles, encoding the P-gp/MDR1 transporter that is implicated in warfarin clearance, whereas no common deleterious variants were identified (Fig. 3b). However, given the controversy regarding the functional impacts of common *ABCB1* variants, such as rs1045642 and rs2032582, future research is necessary to delineate associations between *ABCB1* genotypes and factors related to P-gp/MDR1 activity [21]. Combined, our analyses pinpoint *CYP2C9* and *ABCB1* as loci for which comprehensive NGS profiling can likely reveal substantial additional information regarding the unexplained variability in warfarin dose requirements.

#### Simvastatin myopathy

ADRs related to high-dose simvastatin therapy are strongly linked to the common (12.9% MAF) genetic variant rs4149056 in *SLCO1B1* (encoding the transporter OATP1B1) with an odds ratio of 4.5 per copy of the risk allele [22]. Toxicity is caused by an impaired hepatic uptake of the drug that results in elevated plasma concentrations of simvastatin acid, which have been shown to cause myotoxicity in vitro [23] (Fig. 3d). In our analyses, we correctly predicted the functional impact of rs4149056 and did not find additional common variants with reduced functionality. However, we identified rare deleterious variants with an aggregated frequency of 1.2%, which are estimated to jointly explain 8.7% of the genetic basis of *SLCO1B1* variability (Fig. 3e). Similarly, rare variants are expected to contribute substantially to the metabolism and transport of simvastatin with an aggregated rare functional variant frequency of 1.3, 1.1, and 0.8% for *ABCB1*, *CYP3A4*, and *ABCG2*, respectively (Fig. 3f).

#### Voriconazole efficacy and ADRs

**Fig. 4:**
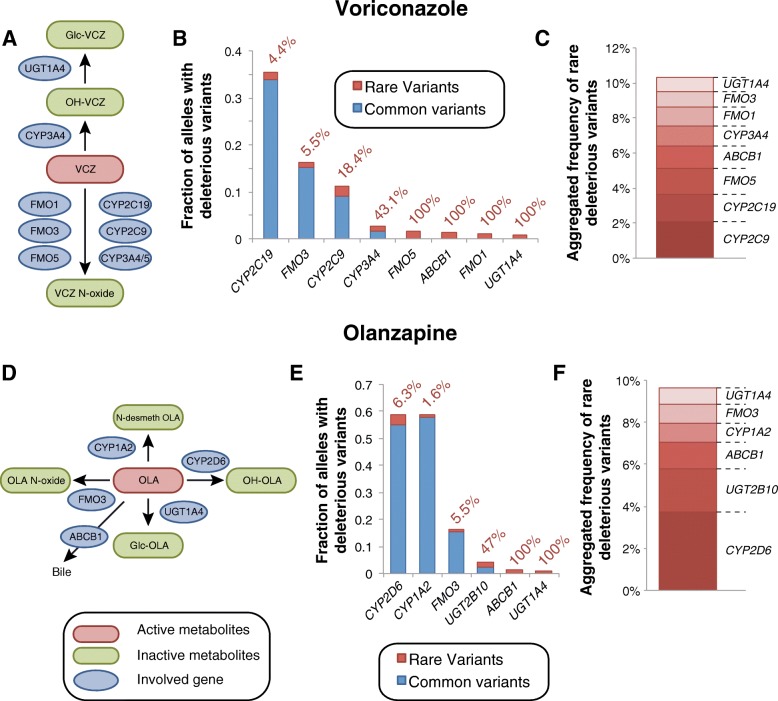
Evaluation of the role of rare genetic variants for voriconazole and olanzapine pharmacokinetics. **a** Schematic depiction of key events in voriconazole metabolism. See www.pharmgkb.org/pathway/PA166160640 for further information. **b** Overview of the aggregated frequencies of common (MAF ≥ 1%, blue) and rare deleterious genetic variants (MAF < 1%, red) in genes involved in voriconazole metabolism. Values next to the columns indicate the relative contribution of rare genetic variants. **c** Stacked column plot showing the aggregated frequency of deleterious rare variants of potential relevance for voriconazole metabolism. **d** Schematic showing steps involved in the pharmacokinetics of the antipsychotic olanzapine. See www.pharmgkb.org/pathway/PA166165056 for further information. **e** Overview of the aggregated frequencies of common (MAF ≥ 1%, blue) and rare deleterious genetic variants (MAF < 1%, red) in genes implicated in olanzapine clearance. Values next to the columns indicate the relative contribution of rare genetic variants. **f** The aggregated frequency of deleterious rare variants of potential relevance for olanzapine metabolism is shown

Voriconazole is a triazole antifungal agent exhibiting large inter-individual variability in serum concentrations that is a common reason for therapeutic failure or the manifestation of ADRs. Voriconazole is extensively metabolized by various CYPs (CYP2C19, CYP2C9, and CYP3A4) and FMOs (FMO1, FMO3, and FMO5) accounting for 75 and 25% of its hepatic metabolism, respectively (Fig. 4a) [24, 25]. Genetic polymorphisms in *CYP2C19* have been reproducibly linked to differences in voriconazole serum levels and jointly explain around 50% of the inter-individual variability in voriconazole metabolism ([26] and references therein). In addition to *CYP2C19* alleles, clinical pharmacogenetic studies also implicated reduced functionality variants of *CYP2C9* (CYP2C9*2) and *CYP3A4* (rs4646437) in differences in voriconazole pharmacokinetics [27, 28]. For *CYP2C19*, our analyses identified rare deleterious variants with an aggregated frequency of 1.6%, whereas the common functional *CYP2C19* alleles CYP2C19*2 and CYP2C19*17 showed frequencies of 18.5 and 15.3%, respectively (Fig. 4b). Consequently, rare variants are estimated to account for 4.4% of the overall genetic variability of CYP2C19 function. Furthermore, rare alleles contributed substantially to the variability in other genes implicated in voriconazole efficacy and ADRs, including *FMO1* (100% contribution), *FMO5* (100%), *CYP3A4* (43.1%), and *CYP2C9* (18.4%; Fig. 4b, c).

#### Serum olanzapine levels

The therapeutic benefits for schizophrenic or bipolar patients when treated with the antipsychotic olanzapine are limited by extensive inter-individual variability in olanzapine serum concentrations, which can result in exposure levels outside the therapeutic interval [29]. As olanzapine serum levels are directly linked to the likelihood of therapeutic success [30] and the risk of ADRs [31, 32], an individualization of dosing regimens promises to increase treatment success rates.

While the metabolism of olanzapine is well characterized, the influence of genetic factors is more controversial (Fig. 4d). CYP2D6 and UGT2B10 hydroxylate or glucuronidate olanzapine, respectively, but so far, no study demonstrated clinically relevant effects of haplotypes of these genes on olanzapine exposure. In contrast, multiple clinical association studies found that genetic variants in *CYP1A2*, *FMO3*, and *UGT1A4* could explain up to 50% of differences in olanzapine serum levels, whereas other studies failed to replicate such associations ([33] and references therein). Rare variants substantially contribute to *UGT1A4* and *UGT2B10* variability and are predicted to account for 100 and 47% of the genetically encoded inter-individual variability in the functionality of these genes (Fig. 4e, f). On the contrary, we estimate that rare variants only explain 6.3, 5.5, and 1.6% of the variability in *CYP2D6*, *FMO3*, and *CYP1A2*, respectively.

#### Irinotecan toxicity

**Fig. 5:**
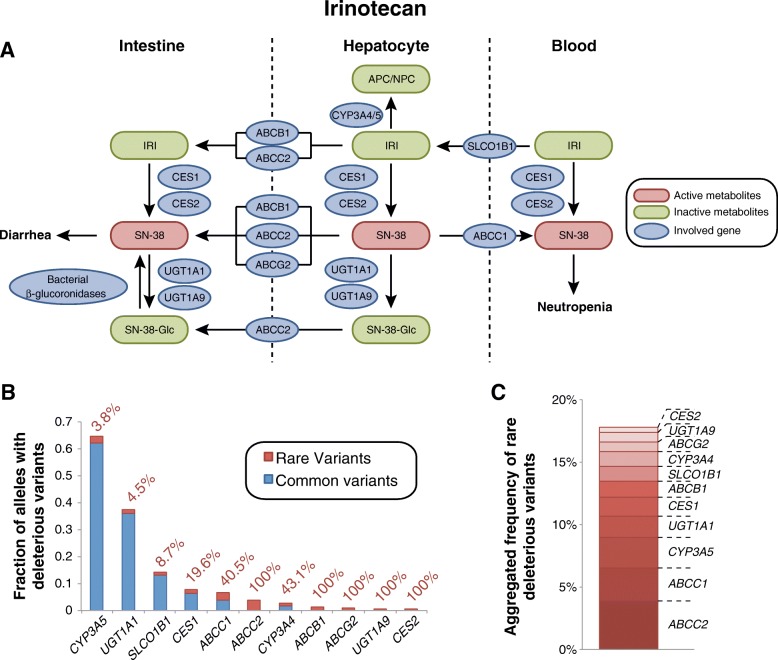
Analysis of genetic factors contributing to dose-limiting irinotecan toxicity. **a** Scheme showing tissue specific involvement of gene products in the irinotecan pathway. See www.pharmgkb.org/pathway/PA2001 for further information. **b** Overview of the aggregated frequencies of common (MAF ≥ 1%, blue) and rare deleterious genetic variants (MAF < 1%, red) in genes implicated in irinotecan metabolism and transport. Values next to the columns indicate the relative contribution of rare genetic variants. **c** Stacked column plot showing the aggregated frequency of deleterious rare variants involved in irinotecan ADME

Irinotecan is a topoisomerase inhibitor prodrug that is used in combination therapy for advanced colorectal, lung, and other cancers. Irinotecan has a narrow therapeutic window and, as a consequence, up to 36% of patients suffer from dose-limiting toxicities [34]. Irinotecan is subjected to a complex interplay of competing metabolic activation and inactivation pathways (Fig. 5a). Around 97% of irinotecan is metabolized by CYP3A4 and CYP3A5 to the pharmacologically inactive metabolites APC and NPC, while only 3% become metabolically activated into SN-38 by the carboxylesterases CES1 and CES2. Subsequently, SN-38 is detoxified by glucuronidation mediated by UGT1A1 and, to a lesser extent, UGT1A9. Irinotecan and its metabolites are excreted into the bile and intestine via multiple transporters of the ABC family. Importantly, the β-glucuronidase enzymes of the intestinal microflora can re-activate glucuronidated SN-38 resulting in diarrhea and damage to all segments of the intestine [35].

Genetic variants in *UGT1A1* have been reproducibly linked to neutropenia and diarrhea toxicity in various ethnicities and dosing regimens [36]. Furthermore, multiple polymorphisms in the transporter genes *ABCB1*, *ABCC1*, *ABCC2, ABCG2*, and *SLCO1B1* have been implicated in irinotecan clearance and/or risk of toxicity [37–40]. While associations between *CYP3A* genotype and irinotecan pharmacokinetics are controversial, incorporation of CYP3A activity data into dosing calculations have resulted in reduced incidence of severe neutropenia [41].

Interestingly, our computational analyses of population-scale sequencing data indicate that rare genetic variants are important factors for irinotecan activation and transport (Fig. 5b). The aggregated frequency of rare deleterious alleles in *CES1* and *CES2* were 1.5 and 0.4%, respectively, accounting for 19.6 and 100% of the functional genetic variants in these genes. Similarly, for *SLCO1B1* and *ABCC1*, 8.7 and 40.5% of all deleterious variants were assigned to rare variants, while for *ABCB1*, *ABCC2*, and *ABCG2*, no common variants with functional consequences were identified. Notably though, we do not consider here variants with functional impacts that do not result in changes of the gene product, such as the synonymous variants rs1045642 (*ABCB1* I1145I) and rs1128503 (*ABCB1* G412G) or the UTR variant rs717620 in *ABCC2*. In contrast, inter-individual variability in UGT1A1 is primarily due to common polymorphisms, such as UGT1A1*28, and only an estimated 4.5% are allotted to rare SNVs.

Combined, our evaluations indicate that rare genetic variants in pharmacogenes have the potential to explain a substantial part of the unexplained genetic variability in drug metabolism phenotypes. Examples were selected for which gene-drug interactions were well studied, and we speculate that the relative importance of rare variants is even higher for less extensively characterized drugs. Furthermore, we give indications about the extent of genetically encoded functional variability that would be missed when only considering common genetic variants, thereby providing guidance for the optimal drug-specific choice of genotyping strategy.

## Discussion

From a drug development perspective, an appropriate pharmacokinetic profile is of key importance to achieve the desired spatial and temporal exposure pattern of a given drug of interest. However, genetic variants in ADME genes encoding for transporters, drug-metabolizing enzymes, or nuclear receptors modulate drug pharmacokinetics and thus impact treatment efficacy and the risk of ADRs. Consequently, 190 drugs approved by the US Food and Drug Administration (FDA) and 155 drugs approved by EMA currently contain pharmacogenetic information in their labels, of which many are related to drug pharmacokinetics [42, 43]. Besides well-characterized common polymorphisms, ADME genes harbor a plethora of rare genetic variants that are not interrogated by current pharmacogenomic genotyping panels. By leveraging large-scale whole-exome sequencing data from 60,706 individuals, we present here the first analysis in which we systematically integrated information about rare genetic variability into predictions of pharmacokinetic variability (Additional file 2: Table S2).

Individual in silico functionality prediction algorithms distinguish deleterious from neutral variants with sensitivities and specificities between 60 and 90%. Furthermore, by using computational methods optimized for the evaluation of pharmacogenes with low evolutionary constraints, we were able to show that up to 92% sensitivity and 95% specificity can be achieved for loss-of-function and functionally neutral variants, respectively [13]. Furthermore, rare copy number variations in pharmacogenes, accounting for up to 1% of all loss-of-function alleles, are an additional source of genetic variability with relevance for drug metabolism phenotypes that are commonly not considered by computational functionality prediction algorithms [44].

Notably, genetic variability in non-coding regions has been demonstrated to have important influence on phenotypic traits [45]. However, while promising progress has been made regarding the prediction of the effects of those variants based on DNA sequence [46], no current prediction framework can reliably predict the functionality of non-coding genetic variation, such as synonymous variants or variants located in UTRs, promoters, or enhancers. With respect to *CYP* alleles, such mutations represent < 1% of all functionally important variant alleles described (https://www.pharmvar.org/genes). We therefore restricted our analyses to the evaluation of LOF variants and variants that directly affect the amino acid sequence of their respective gene products.

Thus, while the predictive power of current functionality prediction methods is still not sufficient to support a recommendation of these tools for genetic counseling of individual patients, our data indicate however that leveraging of NGS technology can yield significant amounts of additional information for pharmacogenomic predictions on a population scale. Accordingly, we advocate for the development of a widened perspective in which conclusions about the functionality of a gene product are not solely based on the interrogation of few common variants. Rather, we recommend that the entire spectrum of genetic variability, including rare or novel variants, should be considered and integrated into gene activity scores. This holistic perspective is especially important as rare polymorphisms are enriched in variants that alter the functionality of the gene product and are the sole genetic factors for variability of more than 50% of the pharmacogenes analyzed (Fig. 2b).

Relating the functional inventory of pharmacogenetic variability to the pharmacology of selected drugs of interest can provide important insights into predicted hotspots of unexplained inter-individual differences in drug metabolism-related phenotypes. In this work, we estimated the relative contribution of rare genetic variants to the variability in pharmacokinetics and/or ADR risk of five clinically important drugs from different therapeutic areas. Rare variants are estimated to contribute significantly to the inter-individual variability of warfarin pharmacokinetics and irinotecan toxicity accounting for 18.4% of deleterious *CYP2C9* alleles and > 40% of the variability in irinotecan transport (Figs. 3a and 5). In contrast, the relative importance of rare variants is expected to be lower for the metabolism of simvastatin, voriconazole, and olanzapine for which rare variants only contribute between 1.6 and 8.7% of the key metabolic and/or transport processes. Thus, we find that the relevance of rare genetic variants is highly drug-specific, depending on the gene products involved. These findings suggest that it is likely that the inter-individual variability in pharmacokinetics and response for certain drugs is to a large extent determined by rare genetic variability, which is important to consider particularly in drug development. Integrating pharmacological information of the drug of interest with information about the distribution of rare variants in pharmacogenes can guide the design of the genotyping strategy most suitable to reveal important additional genetic factors that improve the prediction of drug metabolism phenotypes.

For many drugs, genetic variability cannot be directly translated into effectiveness or ADR susceptibility due to the interdependency of different metabolic pathways. For instance, genetic or pharmacological inhibition of the major pathway of a given drug can result in a shunt to an alternative otherwise negligible metabolic route, as observed for oxycodone [47]. To date, data regarding the effects of such gene-gene or gene-drug interactions is sparse, which complicates predictions of drug effectiveness or safety even when rare genetic variants are incorporated into the analyses. Thus, while the consideration of pharmacogenomic information including rare genetic variants promises to improve ADME predictions, further work particularly in physiologically based pharmacokinetic (PBPK) modeling is necessary to reliably predict treatment outcomes for the individual patient.

Currently, genotyping is largely based on the interrogation of well-characterized, common polymorphisms. This strategy neglects the impacts of rare variants as experimental in vitro or in vivo data that demonstrate their functional impact is not available. Yet, due to rapidly decreasing sequencing times and costs, we suggest that current NGS technology in combination with more advanced computational prediction methods could already today facilitate the refinement of individualized predictions regarding drug efficacy and its propensity to cause ADRs and thereby to contribute to the implementation of pharmacogenetic markers into routine care [48, 49]. Furthermore, the multitude of ongoing sequencing projects on unprecedented scale, such as the 100K Genomes Project run by the British Department of Health and the 1 Million Genomes project as part of the Chinese Precision Medicine Initiative, will soon provide a wealth of information about non-transcribed, regulatory regions and improve linkage information between variants, which will allow to expand the scope of computational analyses from variants to haplotypes. For the field of pharmacogenomics, these developments hold promise to further increase the predictive power of gene-drug response predictions and to allow more accurate estimates of drug response across more narrowly stratified subpopulations.

## Conclusions

We present results suggesting that integration of rare pharmacogenomic variability can improve predictions of drug pharmacokinetics compared to the use of candidate variants. This information is important for drug development and clinical care as well as for future preemptive pharmacogenomic advice. Furthermore, these data incentivize the design of prospective trials using NGS-based genotyping for specific medications, such as warfarin and irinotecan, to assess whether clinical outcomes can be improved.

## Additional files

Additional file 1: Overview of NGS-based analyses of pharmacogenes. (XLSX 42 kb)

Additional file 2: Genes and variants not considered in current drug response predictions. (XLSX 36 kb)

## References

[1] Lauschke VM, Ingelman-Sundberg M (2016). The importance of patient-specific factors for hepatic drug response and toxicity. Int J Mol Sci.

[2] Pirmohamed M, James S, Meakin S (2004). Adverse drug reactions as cause of admission to hospital: prospective analysis of 18 820 patients. BMJ.

[3] Sim SC, Kacevska M, Ingelman-Sundberg M (2012). Pharmacogenomics of drug-metabolizing enzymes: a recent update on clinical implications and endogenous effects. Pharmacogenomics J.

[4] Matthaei J, Brockmöller J, Tzvetkov MV (2015). Heritability of metoprolol and torsemide pharmacokinetics. Clin Pharmacol Ther.

[5] Lauschke VM, Milani L, Ingelman-Sundberg M (2017). Pharmacogenomic biomarkers for improved drug therapy-recent progress and future developments. AAPS J.

[6] Singh RR, Luthra R, Routbort MJ (2016). Implementation of next generation sequencing in clinical molecular diagnostic laboratories: advantages, challenges and potential. Exp Rev Precision Med Drug Dev.

[7] Gordon AS, Tabor HK, Johnson AD (2014). Quantifying rare, deleterious variation in 12 human cytochrome P450 drug-metabolism genes in a large-scale exome dataset. Hum Mol Genet.

[8] Fujikura K, Ingelman-Sundberg M, Lauschke VM (2015). Genetic variation in the human cytochrome P450 supergene family. Pharmacogenet Genomics.

[9] Kozyra M, Ingelman-Sundberg M, Lauschke VM (2017). Rare genetic variants in cellular transporters, metabolic enzymes, and nuclear receptors can be important determinants of interindividual differences in drug response. Genet Med.

[10] Bush WS, Crosslin DR, Owusu-Obeng A (2016). Genetic variation among 82 pharmacogenes: the PGRNseq data from the eMERGE network. Clin Pharmacol Ther.

[11] Lek M, Karczewski KJ, Minikel EV (2016). Analysis of protein-coding genetic variation in 60,706 humans. Nature.

[12] Abecasis GR, Chakravarti A, Donnelly P (2015). A global reference for human genetic variation. Nature.

[13] Zhou Y, Mkrtchian S, Kumondai M, et al. An optimized prediction framework to assess the functional impact of pharmacogenetic variants. Pharmacogenomics J. (In review) 10.1038/s41397-018-0044-2 PMC6462826 30206299

[14] Nelson MR, Wegmann D, Ehm MG (2012). An abundance of rare functional variants in 202 drug target genes sequenced in 14,002 people. Science.

[15] Tennessen JA, Bigham AW, O’Connor TD (2012). Evolution and functional impact of rare coding variation from deep sequencing of human exomes. Science.

[16] Johnson JA, Cavallari LH (2015). Warfarin pharmacogenetics. Trends Cardiovasc Med.

[17] Kaminsky LS, Zhang Z-Y (1997). Human P450 metabolism of warfarin. Pharmacol Ther.

[18] Wadelius M, Sörlin K, Wallerman O (2003). Warfarin sensitivity related to CYP2C9, CYP3A5, ABCB1 (MDR1) and other factors. Pharmacogenomics J.

[19] Pautas E, Moreau C, Gouin-Thibault I (2009). Genetic factors (VKORC1, CYP2C9, EPHX1, and CYP4F2) are predictor variables for warfarin response in very elderly, frail inpatients. Clin Pharmacol Ther.

[20] Klein TE, Altman RB, The International Warfarin Pharmacogenetics Consortium (2009). Estimation of the warfarin dose with clinical and pharmacogenetic data. N Engl J Med.

[21] Hodges LM, Markova SM, Chinn LW (2011). Very important pharmacogene summary: ABCB1 (MDR1, P-glycoprotein). Pharmacogenet Genomics.

[22] Link E, Parish S, SEARCH Collaborative Group (2008). SLCO1B1 variants and statin-induced myopathy—a genomewide study. N Engl J Med.

[23] Kobayashi M, Chisaki I, Narumi K (2008). Association between risk of myopathy and cholesterol-lowering effect: a comparison of all statins. Life Sci.

[24] Hyland R, Jones BC, Smith DA (2003). Identification of the cytochrome P450 enzymes involved in the N-oxidation of voriconazole. Drug Metab Dispos.

[25] Yanni SB, Annaert PP, Augustijns P (2008). Role of flavin-containing monooxygenase in oxidative metabolism of voriconazole by human liver microsomes. Drug Metab Dispos.

[26] Owusu Obeng A, Egelund EF, Alsultan A (2014). CYP2C19 polymorphisms and therapeutic drug monitoring of voriconazole: are we ready for clinical implementation of pharmacogenomics?. Pharmacotherapy.

[27] Niwa T, Hata T (2016). The effect of genetic polymorphism on the inhibition of azole antifungal agents against CYP2C9-mediated metabolism. J Pharm Sci.

[28] He HR, Sun JY, Ren XD (2014). Effects of CYP3A4 polymorphisms on the plasma concentration of voriconazole. Eur J Clin Microbiol Infect Dis.

[29] Patel MX, Bowskill S, Couchman L (2011). Plasma olanzapine in relation to prescribed dose and other factors. J Clin Psychopharmacol.

[30] Perry PJ, Lund BC, Sanger T (2001). Olanzapine plasma concentrations and clinical response: acute phase results of the North American Olanzapine Trial. J Clin Psychopharmacol.

[31] Perry PJ, Argo TR, Carnahan RM (2005). The association of weight gain and olanzapine plasma concentrations. J Clin Psychopharmacol.

[32] Kinon BJ, Volavka J, Stauffer V (2008). Standard and higher dose of olanzapine in patients with schizophrenia or schizoaffective disorder. J Clin Psychopharmacol.

[33] Söderberg MM, Dahl M-L (2013). Pharmacogenetics of olanzapine metabolism. Pharmacogenomics.

[34] Fuchs CS, Moore MR, Harker G (2003). Phase III comparison of two irinotecan dosing regimens in second-line therapy of metastatic colorectal cancer. J Clin Oncol.

[35] Brandi G, Dabard J, Raibaud P (2006). Intestinal microflora and digestive toxicity of irinotecan in mice. Clin Cancer Res.

[36] Campbell JM, Stephenson MD, Bateman E (2017). Irinotecan-induced toxicity pharmacogenetics: an umbrella review of systematic reviews and meta-analyses. Pharmacogenomics J.

[37] Han J-Y, Lim H-S, Yoo Y-K (2007). Associations of ABCB1, ABCC2, and ABCG2 polymorphisms with irinotecan-pharmacokinetics and clinical outcome in patients with advanced non-small cell lung cancer. Cancer.

[38] Han J-Y, Lim H-S, Park YH (2009). Integrated pharmacogenetic prediction of irinotecan pharmacokinetics and toxicity in patients with advanced non-small cell lung cancer. Lung Cancer.

[39] Innocenti F, Kroetz DL, Schuetz E (2009). Comprehensive pharmacogenetic analysis of irinotecan neutropenia and pharmacokinetics. J Clin Oncol.

[40] Li M, Seiser EL, Baldwin RM (2018). ABC transporter polymorphisms are associated with irinotecan pharmacokinetics and neutropenia. Pharmacogenomics J.

[41] van der Bol JM, Mathijssen RHJ, Creemers GJM (2010). A CYP3A4 phenotype-based dosing algorithm for individualized treatment of irinotecan. Clin Cancer Res.

[42] https://www.fda.gov/Drugs/ScienceResearch/ResearchAreas/Pharmacogenetics/ucm083378.htm. Accessed 20 May 2017.

[43] Ehmann F, Caneva L, Prasad K (2015). Pharmacogenomic information in drug labels: European Medicines Agency perspective. Pharmacogenomics J.

[44] Santos M, Niemi M, Hiratsuka M, et al. Novel copy-number variations in pharmacogenes contribute to interindividual differences in drug pharmacokinetics. Genet Med. 2017; 10.1038/gim.2017.156. [Epub ahead of print] 10.1038/gim.2017.156 29261188

[45] Maurano MT, Humbert R, Rynes E (2012). Systematic localization of common disease-associated variation in regulatory DNA. Science.

[46] Lee D, Gorkin DU, Baker M (2015). A method to predict the impact of regulatory variants from DNA sequence. Nat Genet.

[47] Samer CF, Daali Y, Wagner M (2010). The effects of CYP2D6 and CYP3A activities on the pharmacokinetics of immediate release oxycodone. Br J Pharmacol.

[48] Lauschke VM, Ingelman-Sundberg M (2018). How to consider rare genetic variants in personalized drug therapy. Clin Pharmacol Ther.

[49] Lauschke VM, Ingelman-Sundberg M (2016). Precision medicine and rare genetic variants. Trends Pharmacol Sci.

[50] Spitzer M, Wildenhain J, Rappsilber J (2014). BoxPlotR: a web tool for generation of box plots. Nat Methods.

